# Progressive Dyspnea in a 40-Year-Old Man Caused by Giant Mediastinal Thymolipoma

**DOI:** 10.1155/2016/3469395

**Published:** 2016-05-17

**Authors:** Mohammad Vaziri, Kamelia Rad

**Affiliations:** ^1^Minimally Invasive Surgery Research Center, Iran University of Medical Sciences, Tehran, Iran; ^2^Rasoul Akram Hospital, Niayesh Avenue, Sattar Khan Street, Tehran 1448933, Iran; ^3^Iran University of Medical Sciences, Tehran, Iran

## Abstract

Thymolipomas are rare tumors of the anterior mediastinum containing both thymic stroma and an abundance of fat. We present a 40-year-old man with no underlying disease who presented with cough and progressive dyspnea. Clinical evaluation revealed a giant mass of lipid density filling almost the entire left hemithorax with mediastinal shift. Total excision of the 40 × 33 × 8 cm mass weighing 4 kg was performed via a left thoracotomy and the histopathologic diagnosis of the mass was reported as thymolipoma. The patient remains alive and disease-free, twelve months after the intervention.

## 1. Introduction

Thymolipoma is a rare, slow-growing, benign tumor composed of mature adipose cells and thymic tissue. Although it can be seen at any age, it occurs most commonly in children and young adults. They may be identified incidentally during a diagnostic workup for other medical problems and may reach large dimensions and manifest themselves clinically by compression of adjacent structures. Infrequently, a thymolipoma has been associated with a systemic disease state or thymoma. We present a patient with a huge mediastinal mass that proved to be a thymolipoma. The case was presented because of the rarity and gigantic size of a mediastinal mass.

## 2. Case Presentation

A 40-year-old man with progressive dyspnea was referred to our center for evaluation of a giant mediastinal mass. The only other symptom was cough occurring ten months prior to admission. The patient had no underlying disease, no positive family history, and no drug consumption. Clinical evaluation revealed decreased left-sided lung sounds with no adenopathy and organomegaly and no signs indicative of heart compression. Results of laboratory tests were normal as well as cardiac echocardiography and abdominal sonography.

**Figure 1:**
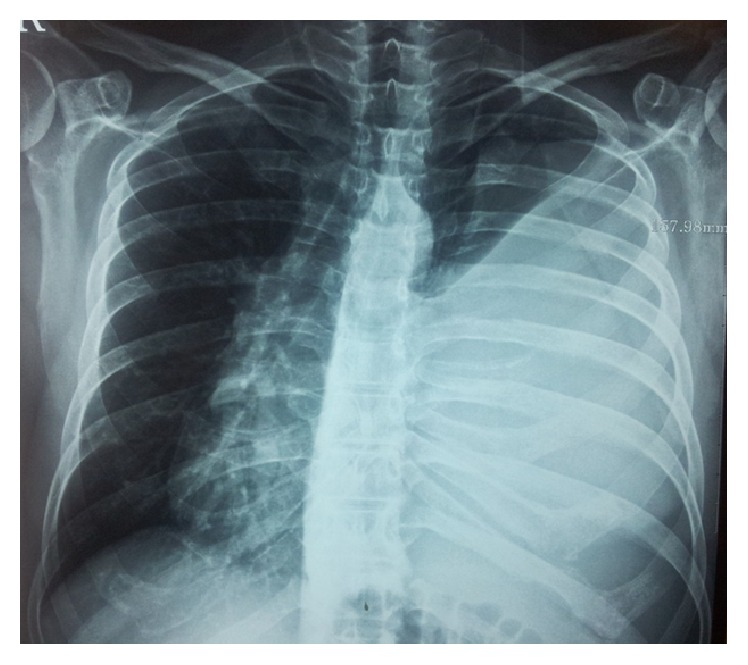
Chest X-ray shows a large left-sided mass density with obvious mediastinal shift including trachea and heart displacement.

Chest X-ray (Figure 1) showed a large left-sided mass density with obvious mediastinal shift including trachea and heart displacement with barely visible lung in the apex and no other lesions in contralateral lung or ribs including calcification, cavity, and nodule.

**Figure 2:**
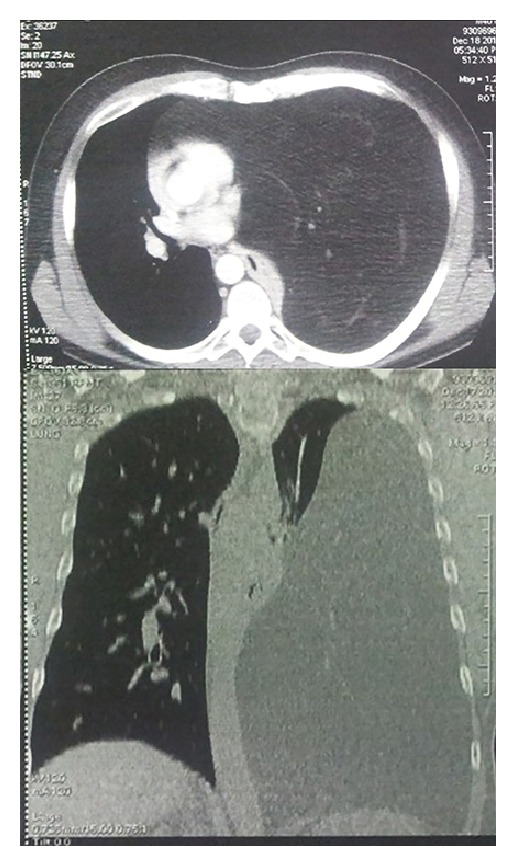
Chest CT scan reveals a huge mediastinal mass with compressive effect on neighboring structures and mediastinal shift.

Chest computed tomography (CT) (Figure 2) showed a large heterogeneous fatty mass in the left hemothorax with mediastinal shift, complete collapse of the lung, left hemidiaphragmatic inferior displacement, and no tissue invasion. The huge size of this tumor made it very difficult to have a correct preoperative diagnosis regarding the exact location or origin of the mass. The case was discussed with pulmonologist and oncologist colleagues and the surgical resection of the mass was recommended with no needle biopsy attempt. We decided not to perform magnetic resonance imaging (MRI) or positron emission tomography (PET) as well as fine needle biopsy aspirate (FNAB) in our patient, as these would not have changed the management plan or the need for surgery in an uncomplicated patient.

**Figure 3:**
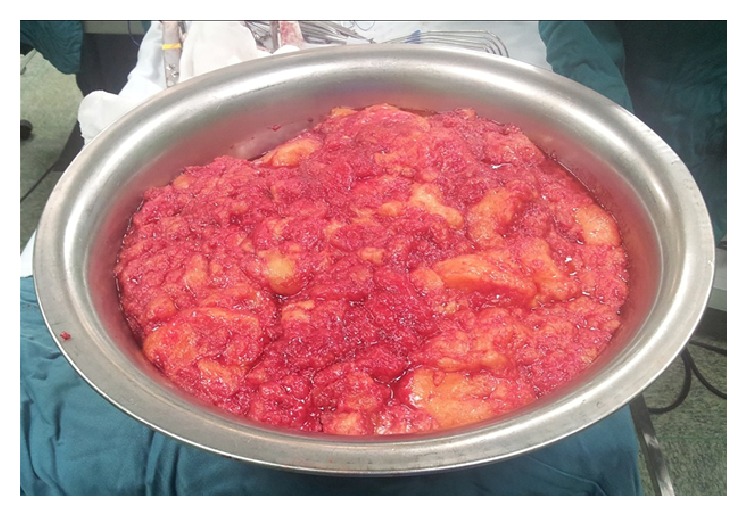
Intraoperative view of a very large tumor placed in a large container.

**Figure 4:**
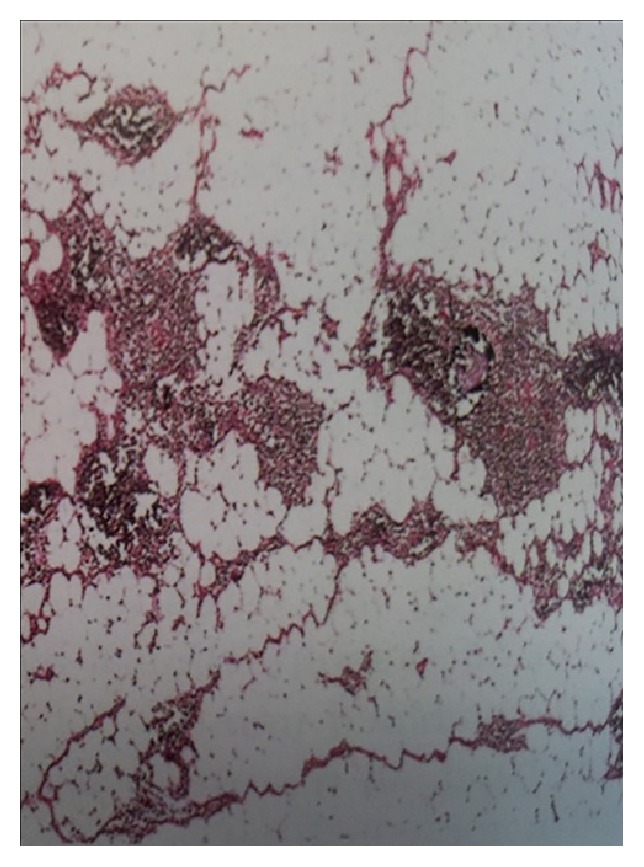
Histologic appearance of thymolipoma showing unremarkable thymic tissue admixed with mature adipose tissue with no evidence of malignancy.

Total excision of the mass was performed via a left posterolateral thoracotomy, although the tumor was too large to be resected as an encapsulated lesion (Figure 3). No pleural fluid and no suspicious mediastinal lymph nodes were observed and no metastatic lesion was detected. Pathologist examination showed a four kg fatty tumor measuring 40 × 33 × 8 cm covered by a thin layer of capsule composed of large lobules of mature fat cells with no atypia and interspersed thymic tissue. Adipose and thymic tissues had no evidence of malignancy (Figure 4).

There was no postoperative complication and the patient remains alive and disease-free, twelve months after the intervention.

## 3. Discussion

Thymolipomas are very rare mediastinal tumors composed of mature adipose and thymic tissue arising in the thymus gland, accounted for 1.1% of all solid mediastinal tumors. Moran and associates [1] reviewed 33 cases of thymolipomas. The ages of their patients ranged from 2 to 64 years, with a mean of 33 years. Regarding clinical presentation, one-half of these patients are asymptomatic, with the lesion being discovered on routine chest radiography. The other half present with symptoms related to compression of the lower respiratory tree. Unusual clinical picture of persistent cervical pain and postprandial nausea and vomiting has also been reported as the presenting symptoms of a thymolipoma [2].

Infrequently, a thymolipoma has been associated with a systemic disease state including myasthenia gravis, Graves' disease, aplastic anemia, red cell aplasia, hypogammaglobulinemia, and lichen planus. None of these were observed in our patient. The literature contains few case reports of thymomas originating from a thymolipoma [3]. Even thymoma containing three histologic subtypes (B1, B2, and B3) simultaneously seen in the same lesion arising within a thymolipoma has been reported [4].

Thymolipoma can be misinterpreted as a pericardial effusion, atelectasis, sequestration, lipoma, or cardiomegaly on radiographic investigations. Chest computed tomography and/or magnetic resonance imaging are usually diagnostic. Characteristically, a thymolipoma presents as a large anterior mediastinal mass that, as it enlarges, spreads into the adjacent visceral compartment. Thymolipomas may adhere to the adjacent structures and displace organs within the chest cavity, but invasion into adjacent structures has not been documented in the literature. Differential diagnoses may include teratoma, lipoma, lipomatosis, and liposarcoma. The latter lesion might have been considered in our patient due to size and induced symptoms of the tumor but there were no weight loss and tissue invasion signs. Although rare, thymolipomas should be considered in the differential diagnosis even in infants presenting with an anterior mediastinum mass. Such a tumor has been reported in a 6-month-old boy [5].

It is rare to encounter thymic tumors including thymoma outside the mediastinum but cases have been described in the neck, trachea, thyroid, lung, and pleura. Patients with pulmonary or pleural thymoma may be asymptomatic or complain of chest pain or breathlessness. Radiologic images show a circumscribed pulmonary or pleural nodule or pleural thickening. Pulmonary thymomas are identical to those that arise in the thymus but are not so clearly encapsulated. However, no thymolipoma in the lung or other mediastinal structures except the thymus has been reported [6, 7]. The huge size of the tumor in our patient made it very difficult to have a correct preoperative diagnosis regarding the exact location or origin of the mass.

Histologically, thymolipomas are composed of large lobules of mature adipose tissue interspersed with islands of thymic tissue. These lesions are benign. The histologic differential diagnosis for thymolipomas includes lipoma, well-differentiated liposarcoma, and thymic hyperplasia. The distinction between a lipoma and a predominantly fatty thymolipoma may be difficult.

Treatment of thymolipoma is surgical excision which is curative and no recurrence or malignant transformation has been reported. Various surgical approaches have been described, including thoracotomy, sternotomy, or video-assisted thoracoscopy [8]. Thoracotomy should be considered for patients with a gigantic mass on CT, as seen in our patient.
